# Neglected ureteral stent in a patient with chronic kidney disease and solitary functioning kidney: case report and review of literature

**DOI:** 10.1097/MS9.0000000000001478

**Published:** 2023-11-07

**Authors:** Mohamed Sakr, Merhan Badran, Prakriti Pokhrel, Ummul Z. Asfeen, Nadin Nouh Badran, Maram Wahed Badran

**Affiliations:** a Alexandria Faculty of Medicine, Alexandria, Egypt; b Kathmandu Medical College and Teaching Hospital, Kathmandu, Nepal; c New York Medical College, Saint Michael’s Medical Center, Newark, NJ

**Keywords:** case report, chronic kidney disease, forgotten double-J stent, neglected DJ stent, solitary kidney

## Abstract

**Introduction:**

A ureteral stent is crucial for managing urinary flow obstruction, ureteral reconstructive surgeries, and iatrogenic ureteral injuries. The authors aim to report a case of forgotten double-J stent for 17 years, the longest time reported in literature in a patient with solitary kidney and no typical long-term complications as stones formation and encrustation.

**Case presentation:**

A 58-year-old male with chronic kidney disease and solitary left kidney presented with left loin pain. With creatinine higher than baseline (6.2 mg/dl), he reported a neglected double-J stent placed 17 years ago in a different hospital. In addition, non-contrast computed tomography and cystoscopy revealed hydronephrosis, cystitis, and an element of infra-vesical obstruction, evident by high bladder neck. Treatment included left percutaneous nephrostomy, but he was readmitted few weeks later for cystoscopy, ureteroscopy, and a new double-J insertion. Due to worsening renal function, he was readmitted four weeks later for cystoscopic removal of the new double-J stent and Transurethral Resection of the Prostate, after which creatinine returned to baseline.

**Discussion:**

Double-J stents are vital in urological procedures, but neglecting their presence can lead to severe complications like encrustations, stone formation, stent fractures, hydronephrosis, infections, and renal function loss. Treatment of long-term complications is difficult and should consider many factors, including the type and severity of associated complications and the patient’s preoperative status.

**Conclusion:**

Preventing complications from neglected or forgotten double-J stents through patient education and follow-up is crucial, especially in those with solitary functioning kidneys due to the imminent loss of renal function.

## Introduction

Highlights

Our case is unique for several reasons:

- Firstly, our patient’s neglected double-J stent endured for a remarkable 17 years, representing the lengthiest duration reported in the literature for a solitary kidney.
- The duration far surpasses previously documented durations in solitary kidneys.
- Secondly, notably, our patient remained free from encrustation or stone formation on the overlooked stent, in contrast to the typical complications encountered in forgotten double-J stent cases.
- This absence of complications distinguishes our case from the majority of reported cases in the literature.

Since their initial descriptions in 1976 by Zimskind and colleagues, double-J stents (DJS) have gained significant importance for various medical procedures, as managing urinary flow obstruction, often caused by stones, fibrosis, or malignancies, and facilitating recovery after reconstructive surgeries and iatrogenic ureteral injuries^1,2^. These stents are available in different lengths and diameters tailored to individual patient requirements. Common initial complications associated with DJS include bladder irritation, spasms, frequent urination, and haematuria. Healthcare providers should replace the stent within 6 weeks to 6 months for optimal performance^2,3^. It is crucial to be mindful of the rare yet serious risks associated with prolonged stent retention, such as stent encrustation, fragmentation, obstruction, urosepsis, and renal failure^4^. Urologists play a pivotal role in educating patients about the importance of post-procedural follow-up, when to replace or remove an indwelling ureteral stent, and possible complications if stent has been overlooked.

In adherence to SCARE guidelines, we present the case of a 58-year-old male patient at Alexandria Main University Hospital, Egypt, who had a neglected DJ stent in a solitary kidney for 17 years^5^. This represents the longest duration reported in the literature without common complications like encrustation or stone formation. This case underscores the management and prevention of this recurrent yet often overlooked phenomenon.

## Case presentation

A 58-year-old male patient presented to our emergency care unit with left loin pain and generalized fatigue. The patient is known to have a solitary functioning left kidney due to right kidney agenesis and has chronic kidney disease with baseline serum creatinine of 2.5 mg/dl. The patient had surgical history of left ureteroscopy (URS) and “stent” insertion 17 years ago at a different hospital. During this presentation, the serum creatinine was measured at 6.2 mg/dl, white blood cell count was normal at 5.79×10^9^/l and no fever was present. An immediate non-contrast computed tomography (NCCT) of the abdomen and pelvis revealed marked hydronephrosis due to a “neglected” DJS. In response, the patient underwent left percutaneous nephrostomy (PCN) insertion, resulting in an initial urine output of 300 ml clear urine. Three days later when renal function tests returned to baseline (2.3 mg/dl), the patient was discharged with readmission scheduled a few weeks later to address the neglected stent.

**Figure 1:**
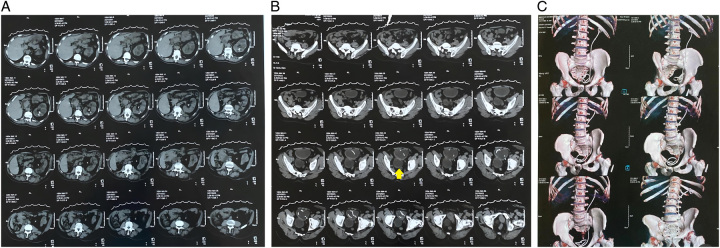
Non-contrast computed tomography (NCCT) abdomen and pelvis showing a non-visualized right kidney and an enlarged left kidney with a percutaneous nephrostomy *in situ*, moderate calyceal dilatation, air foci within the calyces, stranded perinephric fat, and no stones. DJ stent was seen extending from the left pelvicalyceal system (A) into the left ureter, which otherwise had a normal course with no stones, and later into the urinary bladder (B). The urinary bladder was also seen showing a 7 cm diverticulum (arrow), stranded peri-vesical fat reflecting infection (cystitis), and no stones. The three-dimensional reconstructed NCCT showed what would be a course of the existing left ureteral stent extending from the left pelvicalyceal system into the left ureter and eventually the urinary bladder (C).

During this new readmission, fresh renal function tests remained at baseline levels. Another NCCT of the abdomen and pelvis (Fig. 1) was performed, revealing a non-visualized right kidney, an enlarged left kidney with a PCN *in situ* and a left ureter with indwelling DJS without any stones. Additionally, the urinary bladder was identified, showing no stones but a 7 cm diverticulum and stranded peri-vesical fat indicative of infection (cystitis). The left stent was smoothly and uneventfully removed by cystoscopy, which also revealed a high bladder neck and severe pyuria. During this procedure, a diagnostic URS was also performed, demonstrating a widely patent left ureter, left PCN was closed, and a new left DJ stent was inserted and scheduled to be removed 6 weeks later. Postoperatively, the patient’s serum creatinine level measured 2.4 mg/dl. He was discharged with prescriptions for antibiotics and alpha blockers and an indwelling urethral Foley catheter, which was removed 2 weeks later together with the previously closed PCN.

Four weeks later, however, during a follow-up appointment, new renal function results exceeded the normal range, measuring 3.5 mg/dl. Another urethral catheter was promptly reinserted, initially draining 600 cc of clear urine. It was thus deduced that the patient also experienced an element of infra-vesical obstruction that was not alleviated merely by alpha blockers. Consequently, he was readmitted to undergo a transurethral resection of the prostate and removal of the new left DJ stent through cystoscopy.

On outpatient follow-up 1 week later, the patient’s serum creatinine was within baseline range (2.3 mg/dl), and successfully attempted voiding, with no residual urine on post-void ultrasound examination.

## Discussion

An indispensable tool in the urologist armamentarium, DJS are crucial, safe, and effective for a diverse array of clinical indications. During reconstructive procedures, DJS are used to preserve ureter patency, promote healing, and mitigate urine leaks. They are also used to prevent and treat urinary flow obstruction caused by conditions like stones, fibrosis, or cancer^1,2^ and are inserted adjuvant to procedures like ureteroscopic lithotripsy, extracorporeal shock wave lithotripsy (ESWL), and percutaneous nephrolithotomy (PCNL)^6^. However, it is crucial to regularly monitor DJS to prevent severe consequences from neglecting or forgetting them. Complications may include stent discomfort, irritative bladder symptoms, haematuria, bacteriuria, recurrent urinary tract infections, and flank pain. Long-term indwelling DJS can lead to encrustations, stone formation, fractures, blockades, hydronephrosis, infections, and renal function loss^7^.

Many case reports of neglected or forgotten ureteral stents were found upon a review of the literature (Table 1). Our case is unique, however, due to its duration, which is the longest reported in the literature for a single kidney (17 years), significantly longer than durations reported in previous studies by Zanaty and colleagues (3 years) and Azis and colleagues (1 year)^2,22^. In addition, our patient did not experience common complications of long-term indwelling ureteral stents like encrustation or stone formation, similar to a case reported by Tang *et al*.^7^ in which the patient, however, had bilateral functioning kidneys.

**Table 1:** Case reports of forgotten ureteral stents found upon a review of the literature

References	DJ duration (years)	Side	Presentation	Initial indication for DJS	Complication	Management
Tang *et al*.^7^	29	Left	Left flank pain	Removal of ureter stones	HN Hydroureter^c^	URS
Nesbitt *et al*.^8^	26			Reconstructive ureteric surgery during infancy	Encrustation of DJS	
Kim *et al*.^9^	25	Left	Recurrent UTIs Left flank pain LUTS	N/A	Left HN Calcification of DJS Stone formation	LNU
Mahmood *et al*.^10^	15	Right	LAP LUTS Haematuria	Right pyelolithotomy	Encrustation and fragmentation of DJS Stone formation	CLT+ URS LL + 2 consecutive mini-PCNLs
Bidnur *et al*.^11^	12.5	Left	Left flank pain Haematuria LUTS	Left obstructive ureter stone	Encrustation of DJS	PCNL + cystolitholapaxy, + URS
Raina *et al*.^12^	11	Right	Right flank pain	PCNL	Encrusted DJ stent Stone formation	Open cystolithotomy + PCNL + URS lithotripsy
Mejri *et al*.^13^	10	Right	Right LBP LUTS	Hysterectomy	Right HN Calcification of DJS	cystotomy + pyelotomy
Aboutaleb *et al*.^14^	10	Right	Right flank pain burning urination	Ureteric stone removal	Ureter and bladder stones Encrustation of DJS	CLT + URS laser lithotripsy and stent removal
Lee *et al*.^15^	10		Emphysematous perinephric abscess	Obstructive uropathy due to ureteric stricture	Encrustation of DJS Stone formation	Broad spectrum antibiotics modified after C&S + PC drainage + Cystolitholapaxy + Ureterolithotomy
Sohrab *et al*.^16^	8.6^a^		LUTS Haematuria			
Al-Hajjaj *et al*.^17^	8	Right	right flank pain LUTS	Right pyelolithotomy	Right HN Calcification and encrustation of DJS Bladder stones	Open surgery
Zhang *et al*.^18^	6	Right	LUTS	Open ureteric stone removal	Right HN Encrustation of DJS Giant bladder stone	PL via nephroscope and DJS removed with grasper
Kholis *et al*.^3^	5	Right	LUTS Haematuria	Hysterectomy	Bladder stone	CLT
Prihadi *et al*.^19^	4	Right and left	Grossly coming out of patient’s urethra Right flank pain LUTS Haematuria	Myomectomy	Encrusted DJSs	URS PCNL
Kamal *et al*.^20^	4	Bilateral			Encrustation of DJS Stone formation	CLT + bilateral URS + bilateral PCNL
Tao *et al*.^21^	4	Right	Right flank pain LUTS Haematuria	PCNL for right kidney multiple stones	Encrustation and fragmentation of DJS Bladder stone UTI	PCNL with SPC
Zanaty *et al*. ^22^ ^b^	3	Left	Left flank pain LUTS	Single functioning kidney	Encrustation of DJS Stones formation	1^st^ session: mechanical CLT + PCNL 2^nd^ session: USC and new DJS removed in 2 weeks
Al Hajjaj *et al*.^23^	2	Right	LUTS Haematuria	Obstructive calculus	Missed DJS fragment in bladder Stone formation	CLT
Azis *et al*.^2^ ^b^	1		LAP LUTS	Single functioning trans-plant kidney	Stone formation	Mini-PCNL

a Duration of indwelling DJ stent was reported originally in months as 102.9 months.

b These were the reports of forgotten DJ stents in a single kidney.

c In this report, like ours, no encrustation or stone formation were reported as complications of the neglected DJ stent.

C&S, culture and sensitivity; CLT, cystolithotripsy; DJS, double-J stent; HN=hydronephrosis; LAP, lower abdominal pain; LBP, lower back pain; LL, laser lithotripsy; LNU=laparoscopic nephroureterectomy; LUTS, lower urinary tract symptom; N/A, not available; PC, percutaneous; PCNL, percutaneous nephrolithotomy; PL, pneumatic lithotripsy; PN, pyelonephritis;. SPC, suprapubic cystolithotomy; URS, ureteroscopy; UTI, urinary tract infections.

The extent of encrustation is closely linked to the duration the DJS remains in position^2^. Encrustation is also associated with factors as alkaline urine, urinary tract infections, urine composition (specifically, the presence of struvite and calcium phosphate deposits), and metabolic or congenital abnormalities. Lack of urinary acidification has been identified as a significant risk factor for complex stent removal procedures, with urine acidification helping to prevent complications^24^. The material of the stent also plays a role, with silicone DJS having lower incidence of encrustation compared to polyurethane stents^7,12^. We were not able to contact our patient’s previous surgeon to determine the material of his DJS. Moreover, numerous factors as history of stone disease, urinary sepsis, long-indwelling time, chemotherapy, and pregnancy can lead to calculus formation on a stent^16^. The exact cause of stent fragmentation, on the other hand, is unclear, but usually occurs spontaneously after long-indwelling times due to hardening and loss of tensile strength^10^.

The treatment and intervention of forgotten DJS depend on the patient’s preoperative status and the severity of associated complications, including encrustation, stone formation, stent migration, and fragmentation^12^. In most cases, endourological procedures can be undertaken, with few requiring open surgery. However, removing encrusted ureteral stents is a notably difficult process that can result in serious ureteral injury. Slight encrustation can be easily removed with a cystoscope, while large encrustation may require multiple sessions and modalities like URS, ESWL, PCN, cystolithotripsy up to even nephrectomy with reduced renal function^9^. Several modalities can be used in managing nephrolithiasis due to forgotten DJS, with ESWL and URS for small stones and PCNL for large stones. In addition, a recently described modality, mini-PCNL, offers advantages such as shorter hospital stay, reduced postoperative pain, less blood loss, high accuracy in accessing the stone due to ultrasound monitoring, less radiation to staff, and the lack of contrast injection^2^.

The best treatment for these dire complications is prevention by simply educating patients on postoperative procedures and follow-up, ensuring stent replacement or removal within 6 weeks to 6 months^2,3,10^. Various methods, such as register system, wristbands, and mobile apps, have been suggested^7,22,25,26^. Subsequent research ought to prioritize the development of biodegradable DJS material and coating in addition to design alterations to reduce the occurrence of infections, encrustations, and patient discomfort^9^.

Poor socioeconomic, educational, and rural backgrounds are significant factors in overlooking an indwelling ureteral stent. Limited local medical services, transportation difficulties, cost or insurance barriers, poor understanding of follow-up due to language barriers, and male sex are all implicated in forgetting or neglecting DJS^4,6,7,27^. In our patient, these risk factors, including male sex, poor educational and socioeconomic status, lack of health insurance, transportation barriers, and possibly lack of symptoms from chronic complications, were identified.

## Conclusion

Neglected DJS is an alarming phenomenon with severe complications, which can be managed effectively by patient education, follow-up, and addressing barriers to medical care. Special attention should be given to patients with solitary functioning kidneys due to the imminent loss of renal function. Proper history taking, investigations, and management should be tailored to the patient’s preoperative status and associated complications.

## Ethical approval

Our institution does not require ethical approval for reporting individual cases or case series.

## Consent

Written informed consent was obtained from the patient for publication of this case report and accompanying images. A copy of the written consent is available for review by the Editor-in-Chief of this journal on request.

## Source of funding

No source of funding was there for our research.

## Author contribution

All authors contributed equally. M.S.: conceptualization; data curation; investigation; methodology; project administration; writing—original draft; writing—review and editing. M.B.: manuscript preparation; literature review; conceptualization; investigation; methodology; supervision; validation; writing—original draft; writing—review and editing. P.P.: resources; software; supervision; writing—original draft; writing—review and editing. U.Z.A.: writing—original draft; writing—review and editing. N.N.B.: software; writing—review and editing. M.W.B.: software; writing—review and editing.

## Conflicts of interest disclosure

The authors declare that they have no conflict of interest.

## Research registration unique identifying number (UIN)

Not applicable.

## Guarantor

Mohamed Sakr. Merhan Badran.

## Provenance and peer review

Not applicable.

## Data availability statement

Data will be made available upon request.
